# Future physicians’ behavioral intentions towards collaborative practice - a qualitative study on polish final-year medical students guided by the theory of planned behavior

**DOI:** 10.1186/s12909-023-04136-0

**Published:** 2023-03-07

**Authors:** Piotr Przymuszała, Jagoda Szmelter, Łucja Zielińska-Tomczak, Magdalena Cerbin-Koczorowska, Ryszard Marciniak

**Affiliations:** 1 Department of Medical Education, Poznan University of Medical Sciences, 7 Rokietnicka St, 60-806 Poznan, Poland; 2 Students’ Scientific Club of Medical Education, Department of Medical Education, Poznan University of Medical Sciences, Poznan, Poland

**Keywords:** Interprofessional collaboration, Medical students, Theory of planned behavior, Behavioral intentions

## Abstract

**Background:**

Interprofessional collaboration constitutes a vital part of modern patient care, and many of its benefits for patients, medical staff, and the healthcare system have been described. However, little is known about factors influencing medical students’ intentions to work in a collaborative practice after graduation. Therefore, with the theory of planned behavior by Ajzen as a framework, this study aimed to evaluate their intentions and identify factors that influence their attitudes, subjective norms, and perceived behavioral control.

**Methods:**

For this purpose, eighteen semi-structured interviews were conducted with medical students following a thematic guide developed according to the theory. They were thematically analyzed by two independent researchers.

**Results:**

The results showed that their attitudes contained positive (better patient care, comfort and safety of work, learning and development opportunities) and negative examples like the fear of conflicts, losing authority and mistreatment. Sources of social pressure regarding the behavior (subjective norms) involved their peers, other physicians, representatives of other medical professions, patients, and managing bodies. Finally, perceived behavioral control included limited occasions for contacts and interprofessional learning during the studies, existing stereotypes and prejudices, legal regulations and systemic solutions, organizational aspects, and existing relations at the ward.

**Conclusions:**

Analysis showed that Polish medical students generally seem to hold positive views on interprofessional collaboration and feel positive social pressure to get involved in interprofessional teams. However, factors listed in perceived behavioral control may act as barriers in the process.

## Background

The World Health Organization (WHO) defines the occurrence of collaborative practice *“when multiple health workers from different professional backgrounds work together with patients, families, careers and communities to deliver the highest quality of care”* [1]. It is regarded as a vital part of contemporary healthcare systems since no profession can single-handedly respond to all expectations and needs of patients, and its importance is especially emphasized in the case of chronically ill patients or those with complex health needs [2–4]. As it was neatly summarized by Herrmann et al. [5] in their paper, *“the needs of patients are interprofessional, and, thus, improving health care calls for interprofessional efforts.”* Therefore, the collaboration between different healthcare professions seems essential for providing high-quality care for patients [6]. Its benefits and positive patient outcomes are also recognized by healthcare providers and constitute a strong motivational factor for the enhancement of interprofessional collaboration based on mutual respect and effective communication in the healthcare team [4, 6].

Meanwhile, many benefits for patients resulting from the collaboration have already been described in the literature. It has been shown, among others, to improve patient outcomes, safety and satisfaction, decrease the length of hospitalization, reduce medical errors, as well as increase the quality of care [1, 6–9]. For instance, in a prospective, randomized trial by Rich et al. [10], a multidisciplinary intervention was shown to improve the quality of life of elderly patients with congestive heart failure as well as reduce hospital use and medical costs. Similarly, Burns et al. [11] observed greater improvements in outcomes of patients covered by long-term interdisciplinary Geriatric Evaluation and Management program compared with usual care patients, including health perception, social activity, well-being, and life satisfaction. However, not only patients can benefit from interprofessional collaboration. Its positive effects are also felt by healthcare professionals contributing, among others, to increased workplace satisfaction [6]. It also allows for optimizing the use of medical staff, increasing the access to healthcare services for the population [12], which may be of particular importance, especially in countries with a limited number of practitioners like Poland. At the same time, interprofessional collaboration is also considered more financially beneficial as it may reduce the costs of medical errors, for example [12]. On the other hand, the occurrence of collaboration problems might have a negative impact on patient outcomes and work satisfaction, contribute to resource waste or even result in unintentional harm to the patients [2, 8, 13]. In fact, many factors can disrupt effective interprofessional collaboration, and among them, both professional and external ones can be distinguished [8]. Examples of professional factors may include a lack of knowledge, understanding, and trust in representatives of other professions, their roles and skills, while external factors revolve around culture within the profession, limited contact opportunities, or lack of time and remuneration for collaboration [8]. A survey study conducted by Rosenstein and O’Daniel on disruptive staff behaviors showed their negative effects on healthcare team members in terms of concentration, job satisfaction, stress, and frustration, among others, but also on patient outcomes, including adverse events and medical errors, quality of care, patient satisfaction, safety, or even mortality. They also recommended the implementation of strategies to reduce the occurrence of disruptive behaviors. Consequently, it seems vital for members of collaborative practice to share equal responsibility, authority, and decision-making ability [2].

Given the positive effects of interprofessional collaboration in the healthcare setting, its implementation should be supported at the education level and subsequent professional practice [4]. Meanwhile, the understanding of factors that can increase or decrease the willingness to collaborate among members of the healthcare professions seems limited as the existing initiatives to assess its status are rarely located in a theoretical framework. A recent qualitative study on physicians’ and pharmacists’ perspectives on interprofessional collaboration shed some light on the subject from the point of view of Polish healthcare professionals [14]. However, little is known about the intentions of medical students to form a collaborative practice after finishing medical studies. Since the insufficient theoretical location of studies is considered an important problem in the medical education sector [15], we decided to conduct this study using the theory of planned behavior (TPB) by Ajzen [16] as a theoretical framework guided by results of a meta-analysis by Armitage and Conner [17] and other papers [18–20] showing its value in uncovering predictors of healthcare professionals’ behaviors. TPB postulates that the explanatory factor for whether an individual undertakes a given activity is the intention, which in turn is influenced by their attitudes, subjective norms, and perceived behavioral control. According to the theory, attitudes revolve around the perception of the behavior as favorable or unfavorable and the expected outcomes of undertaking it. Subjective norms reflect the social expectations regarding the behavior, while perceived behavioral control involves one’s confidence in the capability to undertake the behavior and control over it [16]. Importantly, TPB allows using the intentions as proxy measures of behavior even when we are unable to measure the actual behavior [21]. However, for this purpose, the inclusion of all three aforementioned elements is necessary. Given the topic of the study, their detailed analysis seems important in predicting medical students’ future behaviors regarding interprofessional collaboration. Consequently, this study aims to utilize the theoretical framework of the theory of planned behavior by Ajzen to evaluate the behavioral intention of Polish final-year medical students to work in an interprofessional collaborative practice after their studies, as well as identify factors influencing their attitudes, subjective norms, and perceived behavioral control.

## Methods

### Researchers’ characteristic

The first author is a physician with a Ph.D. degree and experience with a qualitative methodology. The second author was a final-year medical student at the time of data collection and is now a fresh medicine graduate. The third and fourth authors are pharmacists with a Ph.D. degree and additional BSc in Public Health (Ł.Z-T.) and MSc in Clinical Education (M.C.-K.), which constitute additional assets to the study. The senior author is a physician with a Professor of Medical Sciences title. It should also be added that our research team has experience in quantitative and qualitative research in the area of interprofessional collaboration and education.

### Design of the study and data processing

The study involved semi-structured interviews conducted from February to June 2022 with a thematic guide presented as an interview outline in Table 1, developed following dedicated manuals, including one developed by the author of the theory [21, 22]. Convenience sampling was used because we regarded the opinion of every potential participant as valid, and therefore, we did not want to put any restrictions on their chance to express it. Potential participants were contacted and invited into the study in February 2022 by the second author on the Facebook group of the final-year medical students of our University. They were informed about the objectives of the study and its scientific and voluntary character. The only inclusion criteria for the study were the status of the final-year medical student and the consent to participate in the study. The students were offered no recompense for their participation. Students of the final year were chosen because, nearly at the end of their studies, they have the most thorough picture of the issue under study and the whole education process. Therefore, due to this accumulated experience, they could more precisely identify factors affecting their behavioral intentions, and their insights on the topic could be more comprehensive.

**Table 1:** Interviews’ thematic guide outline

1. Opening question – interprofessional collaboration (IPC) – respondents’ understanding of the concept and previous experiences 2. Attitudes – advantages, disadvantages, positive and negative feelings about IPC 3. Subjective Norms – approval/disapproval of IPC in the environment, sources of social pressure 4. Perceived Behavioral Control – factors enabling, facilitating, preventing, or hindering IPC 5. Current situation in Poland, including possibilities and suggestions for improvement 6. Respondents’ readiness for IPC 7. Closing question – additional issues that the respondent would like to add to the topic	

Taking into consideration the ongoing COVID-19 pandemic and the participants’ safety, the interviews were conducted using the MS Teams application. In order to minimize any inconvenience on the part of respondents from their participation in the study, the interviews were conducted at the time of their choice. Additionally, to avoid pressure on the respondents and ensure their comfort in expressing their genuine thoughts on the topic, all interviews were conducted by the second author, who was also a medical student at the time. Before starting the study, she was trained by the first and third authors on conducting interviews, including the provision of information on qualitative methodology and asking questions, explanation of theoretical backgrounds of the study, question-by-question discussion of the thematic guide, and finally, a mock session of asking questions from the guide. After the interviews, the recordings were encoded and subjected to thematic analysis. It followed the methodology described by Braun and Clarke [23], namely initial familiarization with data, generation of initial codes, themes searching, reviewing them, defining and naming them, and producing the final report. We decided to use thematic analysis to answer our research questions due to its accessibility and flexibility regarding the orientation to data, coding practices, and the development of themes [24, 25]. A theme, according to Braun and Clarke [23], “*captures something important about the data in relation to the research question, and represents some level of patterned response or meaning within the data set*.” In our study, we used a combination of inductive and deductive orientation to the analytic process [24]. It was inductive in the sense that we coded from the data (codes and themes were generated from the interviews with participants without letting our lens “*completely override their stories*” [24]) and deductive as we were also guided by TPB and its variables (attitudes, subjective norms, and perceived behavioral control) to determine what is important or not regarding the research question. The data analysis process was conducted by two researchers to broaden the research perspective and gain greater insight into data [26]. In this process, they concurrently and independently engaged in data familiarization, coding process, and generated initial themes. Then they discussed their findings to define and name the final themes and produce the final report. In this process, they did not aim to meet consensus but share their perspective to gain greater insight into data. It should be emphasized that the authors of the version of thematic analysis used by us explicitly emphasize the incoherence of their approach with practices like measuring intercoder agreement [27], so in our study, we did not use them. The study was reported following the recommendations in standards for reporting qualitative research provided by O’Brien et al. [28]. Eighteen interviews were conducted with 11 female and 7 male final-year medical students. They were between 24 and 31 years old (median: 25). The average duration time of the interviews was approximately 36 minutes. As the concept of data saturation is viewed as challenging or not particularly useful with the adopted approach [26, 29], instead, after discussing this issue, we concluded that the collected data are sufficient to answer our research question.

### Ethical issues

Prior to the study, its protocol was presented to the Bioethical Committee of the Poznan University of Medical Sciences, which confirmed that according to Polish law, its approval was not required (Decision No. KB – 931/21). Still, we paid attention to ensuring the ethical standards of the study in accordance with BERA Guidelines [30]. Before starting each interview, the study protocol was discussed, and informed verbal consent was taken from every respondent for participation and recording of the interview. All respondents were informed about the study’s objectives, the voluntary and anonymous character of their participation, and the possibility of resigning at any moment.

## Results

As a result of the conducted analysis, we generated four themes related to medical students’ narratives regarding their future work in interprofessional collaborative practice. They were discussed below and are also explained in Table 2 in the context of the TPB.

**Table 2:** Four themes generated in the course of the study in the context of TPB

Theme name	Corresponding TPB variable	Relevant elements from the perspective of TPB
Two (or more) heads are better than one – collaborative practice as beneficial	Attitudes	- better patient care - comfort and safety of work - learning and development opportunities
Hoping for best, expecting the worst – collaboration as a threat	Attitudes	- fear of conflicts, being criticized, losing authority, or mistreatment
Meeting expectations – collaborative practice as perceived by relevant others	Subjective norms	- peers - physicians - representatives of other medical professions - patients - managing bodies
When there’s a will, is there a way? – collaborative practice as not necessarily easy to implement	Perceived behavioral control	- limited occasions for contacts and interprofessional learning during the studies - existing stereotypes and prejudices - legal regulations and systemic solutions - organizational aspects at wards - existing relations at the ward

### Two (or more) heads are better than one – collaborative practice as beneficial

The first theme encompasses the way in which medical students regarded their work in a collaborative practice setting as beneficial. The core idea behind this theme is students’ sense of value of collaborative practice, and as many of their utterances seem to correspond closely with the above-mentioned saying, we thought that it might serve as a name summarizing this theme quite neatly.

Better patient care as a result of collaboration was one of such benefits, and students saw patients as primary beneficiaries of interprofessional collaboration between healthcare professionals. The varying educational backgrounds and professional experiences of different professions were seen as a way to provide patients with more comprehensive and better care and reduce the incidence of medical errors. The sense of interprofessional collaboration as a chance for different healthcare professionals (the proverbial two or more heads) to utilize their distinct expertise for patients’ benefit can be traced, for instance, in the following student statements:

R4: *“If we function as a team in the ward and as a team, we approach this patient, not only will the number of views on treating this patient increase, but we will cover a wider spectrum of issues because each team member brings something extra to it.”*

R8: *“they [patients] will be better taken care of in various matters in terms of diet, physiotherapy, and so on. Of course, we try to act on these issues ourselves, but it is known that our knowledge on this subject is limited.”*

Another observation was that members of different professions might sometimes have a closer bond with patients because they spend more time with them than physicians. Consequently, they may observe patients’ everyday functioning at the ward or quickly notice any changes or symptoms. Also, patients were seen as more prone to confide in them than physicians.

R2: *“This is a huge benefit for the patient because the patient will not always tell everything to the doctor because sometimes, she is ashamed. Sometimes she thinks that it is unimportant, and she will get closer, for example, to the midwife [...] or even some things that seem stupid to them they can confide in a nurse, with whom they have more time, I don’t know, to make a joke than a doctor who is in a rush from one place to another, because the nurse can also notice a lot of things when changing, for example, dressings – they are closer to the patient and can inform the doctors.”*

Increased comfort and safety of work constituted another example of the positive influence of interprofessional collaboration. According to the observation that it is impossible for one person (e.g., a physician) to know or be good at everything, respondents viewed their future work as safer and less stressful when undertaken in an interprofessional team and noticed that help from other professionals could also relieve them of some duties and help save time. They also noticed potential improvements in the atmosphere at work. For example:

R4: *“And the greatest benefit of this is, to me, from our medical perspective - the doctor has much more freedom at work. It certainly reduces the stress level because he or she knows that they can trust the competence of other team members.”*

R2: *“this can only have benefits for a simple reason. Firstly, it is the atmosphere, even for the doctors themselves. The atmosphere and pleasure of work and cooperation, i.e., when there are no unnecessary tensions caused by some hierarchy.”*

These aspects were especially important for them in the context of the approaching beginning of their professional careers. An interesting and vivid account in this regard was presented below. It illustrates both the uncertainty associated with entering the profession freshly after graduation felt by the student and the sense of relief and safety coming from being able to count on other team members.

R9: *“as a young doctor after graduation, I will probably feel worse than some patients on my first shift, and I would like nurses and paramedics more experienced than me, so to speak, to show me a little how some things are done [...]. At the beginning of a job as a doctor, let’s be honest, our experience is absolutely zero, and our studies do not prepare us practically for the profession. But I know that other professions, nursing, and medical rescue, are more practical, so at a younger age, they have more competencies than us, so I would also feel safe.”*

Learning and development opportunities were also viewed as a benefit of collaborative practice. The varying knowledge and skills of other interprofessional team members could provide a learning opportunity for the respondents. As mentioned above in their accounts of collaboration benefits on patient outcomes and comfort of work, students seemed to notice the limitations in their knowledge and expertise as future physicians. This realization can also be traced in the below-mentioned quote of a student acknowledging her weak points and how she was previously able to learn from other professions.

R6: *“there are things that I am good at, and there are things that I can learn from other medical professions - also from my previous experience. And it happened more than once that I was able to learn much more from nurses and paramedics than from other doctors, so deriving from each other, realizing that, in a way, we are partners, and we draw from each other. Not everyone is the alpha and omega, a great manager of the ward or their patient, but each of us is a smaller or larger cog [in the machine], and this is my vision that in such a climate of mutual respect and understanding, we can draw from each other what we can mutually offer each other.”*

At the same time, her perspective shows a humility that seems to come with this realization. The collaboration could also lead to the development of physicians in the spirit of humanistic, patient-centered, and interprofessional practice. The opportunities to develop interpersonal and teamwork skills were also brought up.

R4: *“Personally, from the humanistic side - it seems to me that such an approach would be very positive for the development of medics.”*

Overall, the beneficial aspects of collaborative practice referenced in this theme seem to have a positive effect on their behavioral intentions toward it.

### Hoping for best, expecting the worst –collaboration as a threat

The second theme captures students’ perception of collaboration with other professions as something potentially threatening, and this sense of a threat constitutes its organizing concept. In this theme, medical students reference negative aspects of working in an interprofessional team. However, it is worth noticing that they were mostly related to human factors and involved, among others, the occurrence of conflicts or miscommunications within the team, fear that other team members may criticize their knowledge gaps, and co-workers’ personalities – things as one student (R9) noticed which are not characteristic only for a healthcare setting but “*can occur de facto in any work environment, and in any place*”. The risk of blurring physicians’ competencies and losing authority within the team was also mentioned, and both factors seemed to be closely related. Moreover, these negative influences were often influenced by students’ observations during their studies or accounts of other doctors, as evidenced by the examples below.

R2: *„I am afraid of undermining, for example, my competencies in front of the patient because I had witnessed many times when there was a young resident and a nurse questioned her competencies (an older nurse). […*], *and the nurse can be right over the doctor because, for example, she has been in the profession longer, she has seen things, she has worked with many specialists. It is just pointing things out to the doctor in front of the patient does not bode well”.*

R10: *“this can lead to the so-called getting walked all over, which is often mentioned by older doctors, in particular, to be careful that nurses do not have too much to say because then you will not be able to make independent decisions. And I think that, in some way, there’s a certain risk that if there’s too much dialogue, it might lead to less personal decision-making.”*

Some negative emotions were also associated with the fear of being mistreated by members of other professions, which was influenced by individual students’ own previous poor experiences. An account of the next student describes a phenomenon that resembles some kind of self-fulfilling prophecy in this regard, where the way that other staff members treat medical students (regarded as future physicians) to show them how bad it feels may turn out to be the reason why the same students will have negative and even avoidance attitudes towards collaboration with this profession.

R2: *„very often we met with negative attitudes from nurses or midwives towards us as students [...] where we were pushed around by nurses, who many times even to me personally said that the point is for us to learn how it is because then I won’t be so and so. But as a matter of fact, I did not intend to be so and so, and because they humiliated me on some holiday internships, well, that was a negative experience.”*

What is noteworthy about this theme is its contrast with the previous one, where students presented positive attitudes arising from the abundance of benefits that their work in collaborative practice may bring. However, it seems that students’ sense of threat or associated negative feelings covered by this theme do not necessarily steam from the concept of collaboration but rather its implementation in practice and interpersonal factors. Therefore, we also tried to reflect this in the developed name of the theme, which summarizes how despite generally positive attitudes, some students may fear collaboration. This theme covers aspects that may negatively affect students’ intentions for interprofessional collaboration.

### Meeting expectations – collaborative practice as perceived by relevant others

This theme covers students’ observations about the expectations that other people or groups may have regarding their involvement in interprofessional collaboration. This core idea and the way students may feel pressure from people they consider relevant to meet these expectations guided the decision to develop and name this theme.

One such group for medical students was their peers (other medical students and freshly graduated physicians), who, given the perceived advantages of interprofessional collaboration, were viewed as mostly having positive attitudes and approving of participating in it.

R9: *“It seems to me that most students, at least at the stage of the fifth and sixth year, have a little fear in their eyes, they know that they know something, but well, not that much, [ … ]so it seems to me that in the vast majority, these are young people, young doctors who are just starting and need a sense of security at work and a little bit of showing what’s what and so on.”*

According to the respondents, their positive feelings were especially apparent among those who were perceived to have such qualities as empathy, social skills, openness, willingness to work with people, and humility, among others. They were also viewed as more likely to have a history of contact with students of other faculties, study at other faculty before pursuing medicine, or be actively involved during their studies in the student government, student scientific clubs, organizations, volunteering, or participating in student exchanges, e.g., ERASMUS program. These activities and contact with students of other faculties associated with it were seen as a way to prevent students from living “*in this bubble of our faculty*” (R4) and facilitate making future collaboration decisions.

R3: *“people who were previously involved in such activities and are socially involved because if they get involved at the university in various [student] organizations, scientific clubs, and so on, then you have contact with students of various faculties. So, I have the impression that such people seem to see a greater need for collaboration.”*

As representatives of their future profession, physicians were also an important reference group for the respondents. However, their opinions on the topic varied. Some believed physicians would approve of the behavior, especially those overworked, younger, or less experienced, on account of previously listed benefits of collaboration.

R2: *„I think that the groups that can praise it are the overworked doctors because if they cooperate with nurses, the nurses will relieve them at work [...] and thanks to them there is often no unpleasantness in the wards, mistakes and so on because they think together with the doctor.”*

On the other hand, those physicians who are overconfident in their knowledge and skills or *“on the top of the pyramid,”* as R6 neatly described them, may be less interested in interprofessional collaboration as it may be perceived as diminishing their authority.

R4: *“Older generations of doctors, most likely, are and would be opposed because they are brought up in this authoritarian-pedestal system [...] they would not like it because they base their work on this authority resulting from the institution that is the doctor in their eyes.”*

Representatives of other medical professions were generally believed to hold positive attitudes and approve of the respondents’ involvement in interprofessional collaboration. However, similarly to the group of physicians, older generations were seen as potentially less prone to introducing changes.

R4: *“I’m sure the younger members of the rest of staff would be happy with this, but also the generational divide is important because of the difference between the nurses of this new school [nursing studies], who [ … ]are brought up in a slightly different thinking pattern, and these nurses from post-secondary [vocational] medical schools with a dozen or so years of work experience, [ …*] *based on the principle that we do what the professor did 20 years ago, then they definitely would not satisfied with this, because interprofessional collaboration is also associated with greater responsibility.”*

Patients were also seen as a group that would approve of the behavior, which seems consistent with their potential gains from it. However, there were doubts about whether they would be aware of the benefits resulting from collaboration between different professionals.

R11: *“It seems to me that patients - when they have many people around them, who are each a specialist of some kind, they feel taken care of.”*

On the other hand, as some students noticed, in view of the under-financing of the Polish healthcare sector, the entities who would have to secure finances for the introduction of interprofessional collaboration on the broader scope (e.g., raises in employees’ salaries due to increased duties) were seen as disapproving of the behavior.

R4: *“In our underfunded system, the management of the hospital or, in general, the administrative and accounting department would have a big problem with this because to implement it sensibly, raises for nurses and paramedics would be required. Well, if we want to delegate greater competencies to them, then it is natural that we have to pay them more, and to pay them more, this entity would have to find the money somewhere.”*

R11: *“ I don’t know, I can’t really imagine such a situation [that someone would disapprove], unless someone who has some additional financial costs due to it, I don’t know, in the hospital.”*

Overall, this theme illustrates that Polish medical students feel generally positive social pressure towards interprofessional collaboration as most people relevant to them were seen as approving of the behavior. However, instances of negative pressure may also occur, according to students, and this possibility should be taken into account.

### When there’s a will, is there a way? – collaborative practice as not necessarily easy to implement

The last theme developed in this study describes whether students, despite their generally positive attitudes and sense of social expectations toward interprofessional collaboration referenced in previous themes, feel capable of enacting it. The core organizing concept behind this theme is, therefore, students’ sense of control over the behavior and barriers they may encounter.

Among different factors contributing to this theme, medical students participating in the study described, for instance, the isolation as students of one faculty tend to spend time with each other and how they had limited occasions for contact with students of other healthcare faculties during their studies. They were mostly limited to private contacts and random situations, which seemed to negatively influence their control of the behavior. A comment from one student presented below can serve as a good example of this, as it also shows student’s strong feelings associated with that.

R4: *“It is very painful for me that I have contact with members of other professions through a flat with roommates from our university or Tinder because there, in fact, this exchange between the students takes place the most widely. And during the studies, the university practically does not give any area for establishing cooperation.”*

Although, as students admitted, some elective interprofessional classes were offered for them to choose from (e.g., between medical and pharmacy students), this was deemed unsatisfactory. Students expressed a wish for more interprofessional classes and to make them a standard part of their curriculum. They wished for more communication and simulation classes on the topic, bigger integration between different faculties, and occasions to learn about the roles and competencies of different professionals and their expectations of the physician. These students’ expectations can be observed in the following examples:

R7: *“Certainly, at university, there should be more emphasis on its learning, and if you look at the last twenty years, it’s still better than, let’s say, twenty years ago when there were no such classes at all, but you can definitely have more of them and shorten some parts of those classes that are pointless and spend more time on how to talk to the patient, how to talk to other medical groups and so on and put more emphasis on it. Also, outside of classes, emphasis on integration between faculties [ …], which also develops relationships and soft skills and understanding.”*

R8: *“Maybe we should also have some meetings with dietitians, physiotherapists, and nurses at the beginning of our path so that they tell us what they expect from us or what problems they encounter on a daily basis, or what we do not know and we should know, for instance - it would certainly be easier for us then.”*

The value of such contacts can be evidenced by the account of one of the students about the summer internship:

R9: *“I benefited a lot from vocational internships after the second year [at the emergency department], when, paradoxically, a young paramedic took care of me and showed me some things. [ … ]this experience was good for me when it comes to perceiving and willingness for later collaboration because I also saw how much they [paramedics] do.”*

Another factor mentioned by students that would make it difficult for them to implement interprofessional collaboration behaviors in their future work was the prevailing stereotypes and prejudices about each other among different professions or, as one student (R7) called it - pigeonholing. As one student put it nicely:

R6: *“I think that there is definitely a lack of understanding and stereotypes because we all have a lot of prejudices against each other because doctors are like that, nurses are like that, and paramedics are some other way, and wherever you go to any ward, you hear some opinions generalizing other professions that are often harmful because they build a negative image of these people in us, which is often untrue. And then, entering the profession, we also often function in these prejudices or stereotypes, and we are surprised when we suddenly meet someone from another profession who is cool - it is absurd. [ …] It is also a result of a certain evolution of these professions - some time ago, this profession looked completely different. For example, nurses were after [post-secondary, vocational] school, not after studies. And especially among the senior staff, it lingers somewhere, and then we enter it, duplicate it, and that is where our prejudices and lack of understanding come from.”*

The above observation of the student carefully examines this problem pointing out several important aspects associated with it. Firstly, it describes how stereotypes and prejudices about other professions can distort their perception to the point where collaboration is difficult to achieve. Secondly, the last sentence shows how they can be transferred from generation to generation if not acted upon. And thirdly, it shows their mutuality, meaning that other professions may hold negative views about physicians, which can hinder the relationship even if students want to establish it. This last point is also visible in the quote of a student personally affected by such a barrier.

R3: *“I also take part in courses for physiotherapists, so I also see a sudden barrier when they find out that I am studying medicine, not physiotherapy, and then there is a completely different conversation and a barrier that I do not understand.”*

The next factor revolved around the existence of legal regulations and systemic solutions for interprofessional collaboration, and in the case of our respondents, the lack thereof was also seen as a barrier. The need for clear specification of mutual roles and competencies was noted, along with the ways of establishing such contacts.

R4: *“Collaboration enters in a typical Polish way, i.e., through the back door, from the bottom up, without any regulation or systemic view [ …*]. *These changes in the laws are needed to expand competencies, but this is only one cog in this complex machine, and the main element is certainly a change of outlook and a change of a certain image of hospital treatment. It is definitely a process that takes time.”*

R7: *“[Interprofessional collaboration in Poland] develops naturally [...]there are no, at least at my current state of knowledge, structures, systemic solutions here. It rarely happens from the top, for example, from the director through the heads of departments and so on. [ … ]It is not fully managed in a systemic way.”*

The current organization of wards was also viewed as not completely supporting the implementation of interprofessional collaboration. Students listed aspects like divergent work schedules (working hours) between professional groups, space separation between them, or insufficient personnel numbers due to limited finances and their number at the market. Mentioning them, students also seemed to emphasize their complete lack of understanding and acceptance of such organizational limitations. For example, it can be traced in the extracts from the below comments, where students call them ‘stupid things’, ‘small things’, or ‘rigid division’.

R4: *“There are also such stupid things, like the alignment of work schedules. It may seem quite funny, but in a way, it very much destroys this cooperation that there are such small things, like the inequality of these schedules”.*

R7: *“Such frictions between groups, not necessarily resulting from specific personal factors - for example, that nurses’ duty rooms are far away from medical ones [...] well, there is such a rigid division as if they could not be at least connected by some room, or something like that, or at least close to each other.”*

The respondents also paid attention to the habits and rules they could encounter in their future workplaces. They admitted that it would be much more challenging to initiate interprofessional collaboration in case of a tense atmosphere at the ward and negative attitudes or comments from co-workers. Bigger integration of the personnel and removal of some of the above-mentioned organizational barriers were proposed as a way to bring the healthcare team members closer.

R2: *„[…] most of all, the atmosphere at work makes it possible to make such interpersonal contacts […] If relations are tense between the medical, nursing, and midwifery staff, it will be difficult to enter into such relationships because you feel in advance an attitude that yet another person came who, for example, like doctors from a given ward, does not want to talk to us, and will treat us badly.”*

The next comment additionally shows the barriers students may encounter at their future workplaces following their attempts to introduce innovations.

R6: *“The general atmosphere at work - what was the team like before? How did they work together before? If you are to enter - What are the rules or the broadly understood tradition of the ward or the hospital? Because they will tell you that it has always been this way, it will never be otherwise. It is all such a legacy of the place you come to [ …].*”

Overall, the instances covered in this theme seemed to negatively affect students’ behavioral intentions and act as significant barriers in the process.

## Discussion

In TPB, attitudes towards a given behavior constitute its overall evaluation, including its expected outcomes and associated positive or negative beliefs [16]. For our respondents, one of the advantages of interprofessional collaboration was *better patient care* as a result of it, including its improved comprehensiveness and quality of services, increased patient safety and satisfaction, and reduced number of errors. These students’ observations seem coherent with many studies showing enhanced patient outcomes due to interprofessional collaborative practice and its importance for a safer and more patient-centered care delivery [6–9, 31]. Also, the World Health Organization, in its definition of collaborative practice, recognizes as its main objective the delivery of the highest quality of care [1]. Our respondents recognized that physicians’ knowledge is not unlimited, and interprofessional collaboration would allow them to utilize the knowledge and experience of other healthcare professionals for patient benefit. On the other hand, they also recognized differences in the knowledge about patients and ease of contact with them among different medical professions. Similar observations were made in a study by Salberg et al. [32], where medical and nursing students recognized differences between professions in this aspect, with physicians being regarded as more formal and nurses more caring and having better knowledge of patients than physicians. A more holistic approach of nurses and their better knowledge of patients due to the time spent with them and the utility of using it in the decision-making process to improve patient care was also noticed by medical students in the study by Friman et al. [4].

*Comfort and safety of work*, including less-stressful conditions and improved working atmosphere, were also viewed as benefits of interprofessional collaboration. It seems that for our respondents, this safety of work was associated with avoiding medical errors and, therefore, malpractice suits. It was important, especially in the context of their approaching medical careers. The increased comfort of work seems consistent with reports of the positive influence of interprofessional collaboration on workplace satisfaction [33]. *Learning and development opportunities* for our respondents were a consequence of the varying knowledge and skills of other healthcare workers and, as a result, the opportunity to draw from them to increase their own competencies. The development of interpersonal and soft skills and a more patient-centered approach were also given in this context. Improved interprofessional competencies and knowledge sharing were also among the values, norms, and goals of the interprofessional collaboration initiative in the study by Vestergaard and Nørgaard [33].

However, despite the above-mentioned positive beliefs, some risks associated with *fear of conflicts, losing authority and mistreatment *were also noticed. An interesting observation in this context was made by Friman et al. [4]. In their study on interprofessional collaboration in wound care, medical students expressed fear of not appearing knowledgeable on the topic in front of nursing students. As the authors noticed, this fear might cause some of them to refrain from collaboration in the future to hide their knowledge gaps. They also noticed a strong desire, especially among medical students, for a clear definition of professional roles, the need for each group to adhere to them, and the risk of blurring professional boundaries [4], which was also visible in our study. The concept of equal importance and contributions of different professions may be frowned upon and seen as a threat to professional identity and boundaries by some professionals [34]. A recent qualitative study from Poland also shows physicians’ fear of losing competence and being judged or criticized by other professions for their potential lack of knowledge [14].

Subjective norms take into account the social pressure and expectations perceived by respondents to perform or cease from performing a given behavior [16] and, therefore, require the identification of groups of people as potential sources of such pressure as well as whether they would approve or disapprove of the behavior. Among them, respondents believed that, given its advantages, their *peers* (other medical students and fresh graduates) mostly held positive attitudes toward interprofessional collaboration. The intensity of this belief seemed to be additionally strengthened by their perspective of soon entering the job market. Meanwhile, other studies on medical students show their less positive attitudes towards interprofessional collaboration in comparison with pharmacy [35–37] or nursing students [4, 38], for instance. The study by Wilhelmsson et al. [38] also pointed to the potential influence of other factors that should be examined to understand students’ readiness for interprofessional collaboration giving their personality as an example. Our respondents listed potential factors increasing the willingness for interprofessional collaboration, including specific personality traits and opportunities for previous contacts with students of other faculties like student organizations, volunteering, or student exchanges. This seems to find support in a study, which on the example of the ERASMUS program, shows how participants could observe the organization of healthcare in other countries and, among others, the involvement of other team members in patient care *“who take over a certain part of the duties of doctors (which in Poland have to be performed by doctors alone or are not performed by anyone)”* as one respondent neatly put it [39].

As their future professional group, *physicians* also formed an important point of reference for respondents. Seselja-Perisin et al. [35] observed that physicians’ attitudes toward interprofessional collaboration in their study were more positive than among medical studies, which they attributed to the bigger awareness of its benefits due to their professional experience. However, in the case of our respondents, their views were more mixed with some believing that on the one hand over-worked or less experienced might find the solution favorable while on the other hand others, especially older physicians accustomed to the hierarchical system might oppose it. Medical students in the study by Friman et al. [4] made identical observations, describing differences in the notion of hierarchy and status between younger and older physicians, with the latter perceiving nurses’ role as performers of their recommendations. The notion that younger generations might be more open toward collaboration was also shared by Polish physicians and pharmacists [14]. The next group mentioned by respondents in our study were *representatives of other medical professions* who were mostly believed to be favorable towards interprofessional collaboration. As in studies on students, previous research also shows less positive attitudes among physicians than among other professions [2, 35]. The reason may be that, as it has been noted in the context of these differences, while, for instance, nurses from the beginning are prepared for working in teams, physicians’ learning environment is more independent and competitive [4]. However, as our students noticed, similarly to the group of physicians, the older generation may be less interested in interprofessional collaboration, especially if it would entail additional responsibilities.

Due to the benefits of interprofessional collaboration, *patients* were seen as a group approving the behavior. As mentioned above, it can contribute to more comprehensive patient care, improved treatment quality, and lower mortality rate and incidence of errors, among others [1]. *Managing bodies*, on the other hand, were seen as less favorable towards it, especially if it would involve the necessity to secure additional financial resources like salary raises due to the increased scope of duties of other healthcare professionals. In this context students referred to the underfinancing of the Polish healthcare, which was also noticed in the State of Health in the EU report, with the health spending in Poland as well as the numbers of practising nurses and physicians described as among the lowest in the EU [40]. It is also coherent with a previous study on Polish healthcare practitioners, who admitted the role of policy-makers in influencing their behavior but simultaneously noticed the low interest of the physician self-government or the national insurer in interprofessional collaboration and thus low sense of social pressure coming from them [14]. On the other hand, in the study by Vestergaard and Nørgaard [33], stakeholders expressed positive attitudes towards interprofessional collaboration in the hope that it could increase efficiency and minimize work duplication, among others. It could seem, therefore, that the less positive student perception of attitudes among managing bodies might result from the insufficient promotion of the benefits of interprofessional collaboration in society, including the savings it can bring.

Finally, perceived behavioral control of the behavior encompasses a given person’s ability to perform it, including their confidence and control over the behavior with the role of both situational and internal factors [16]. *Limited occasions for contacts and interprofessional learning during the studies* were mentioned in this context as one of impeding factors. Interprofessional education was defined by WHO as *“occasions when two or more professions learn about, from and with each other to enable effective collaboration and improve health outcomes”* [1]. Its crucial role in improving, among others, the satisfaction and health outcomes of patients, satisfaction and retention of employees, as well as efficiency and cost-effectiveness of the healthcare system, has also been documented [34]. However, as our respondents noticed, the opportunities for interprofessional learning in Poland are limited to elective classes [41], while most contacts with other professions usually occur outside the classes. Meanwhile, such early interprofessional contacts and experiences seem to have a big impact on students’ willingness for active involvement in an interprofessional team [1]. Unfortunately, as studies from other countries show, integrated interprofessional learning and socialization opportunities are still rare in medical universities due to the shape of their curricula focused on meeting needs and transferring knowledge and skills specific to a given profession [3, 5]. This, in turn, contributes to students’ inadequate knowledge and trust in other professions’ roles and their limited ability for teamwork within an interprofessional environment after finishing their studies [3, 5]. As studies show, this lack of knowledge, skills, and negative attitudes can be improved by appropriate educational measures [42]. Our students also noticed the need to learn more about the competencies of other professions. Meanwhile, medical students in the study by Woermann et al. [43] presented low knowledge of other professions’ education and seemed to be aware of it, which was explained by the authors by their focus on their own career as contrasted with nursing students who were viewed as giving more consideration to other professions and had some occasions for contact with them. As previous studies suggest, low knowledge about competencies of other professions and collaboration possibilities may lower the willingness to establish collaboration [14]. The situation is exacerbated when a traditional hierarchy is still present, with physicians viewed as having a superior role [44]. Geographical and location barriers can also occur. For example, as Herrmann et al. [5] describe, in Switzerland, students of medical and other healthcare faculties attend different educational institutions and have limited contact with each other. Recently, a need for restructuring the shape of medical and healthcare curricula has been suggested with a change in focus from a profession-based to an expertise-based care model, which could foster students’ perception of the importance of interprofessional collaboration as well as improve their respect for other healthcare professions [45].

*Existing stereotypes and prejudices* can also act as a barrier to interprofessional collaboration. As a result of the above-mentioned hierarchical structure of healthcare systems, which is still noticeable also in Poland, professionals higher in this hierarchy might have tendency to separate themselves from other professions and stereotype them, which may be present already among students and influence their views on interprofessional collaboration [4, 46–48]. This is supported by the results of Prentice et al. [12], where such stereotyping was demonstrated in the theme ‘the great divide,’ and increased interaction opportunities were suggested to overcome it. Also, another recent study still shows the existence of stereotypes and hierarchy in the division of power among students, with physicians viewed as decision-makers and nurses as assistants [32]. Khalili et al. [34] linked this process to the social identity theory stating that the identification with a given profession leads to a profession-specific cognitive map and orientation towards that profession, and therefore a risk of favoritism and trust towards own profession with bias and distrust towards other professions. This might disturb interprofessional collaboration. Meanwhile, early contact among students and interprofessional learning can also contribute to overcoming such stereotypical views [49]. What may be important, especially in institutions or countries with limited resources, a basic interprofessional course dedicated to overcoming stereotypes does not necessarily has to generate high costs [50]. However, it should also be emphasized that mere contact between different professions is not enough, and according to Allport’s intergroup contact theory simultaneous fulfillment of four conditions is necessary - team members’ equal status, their common goals, cooperation within the group, and the support of authorities in the institution [51]. Moreover, as the study by Yu et al. [52] shows, in contrast to improvements in students’ attitudes toward interprofessional learning and their self-competency, their perceptions of the roles of other professions may be more difficult to change during a single short intervention, which calls for increasing the intensity of occasions for interprofessional learning in the curriculum, including clinical practice. On the other hand, informal contacts between different professions’ representatives seem to allow them to overcome the existing stereotypes [14], which is mirrored by examples provided by respondents in this study.

The existence or lack of *legal regulations and systemic solutions* was also identified as an important factor by the respondents. A similar necessity for precise legal regulations was also previously noticed by Polish physicians and pharmacists [14]. Given that the functioning of the healthcare system and professional competencies are regulated by the appropriate legislature, the presence or lack thereof has an influence on the shape of interprofessional collaboration [14, 53]. *Organizational aspects at wards* involved factors that might hinder the full collaboration, like isolation between different professions in terms of occupied space (e.g., separate rooms for doctors and nurses) and work schedules. Similar isolation was also described by Polish physicians and pharmacists, who additionally viewed collaboration between them as going beyond their usual scope of practice and expected extra space and gratification for that [14]. Also, Muller et al. [54] noticed that organizational factors might impede interprofessional collaboration in rehabilitation clinics providing examples of work overload, or insufficient time and remuneration. Furthermore, *existing relations at the ward* could also be a barrier, including whether interprofessional collaboration would be already established in their future workplace, and the general atmosphere and relations among the rest of the staff. In the study by Friman et al. [4] students also felt that different structures, traditions and care culture within their future practice, including old patterns or authoritarian hierarchies, may be obstacles in this regard. They saw them as controlling factors, which may be easily passed on, and described their experiences with expectations to act as leaders and give orders.

The strengths of this study include its strong theoretical foundation in the theory of planned behavior by Ajzen and an under-researched topic concerning medical students’ behavioral intention to work in interprofessional collaborative practice, including their attitudes, subjective norms, and perceived behavioral control. However, its limitation that should be mentioned is the fact that students who agreed to participate could have more positive opinions on interprofessional collaboration than their colleagues who were not interested in the study. We tried to minimize this risk by emphasizing during the recruitment process that all views are valuable, and we did not impose any other inclusion criteria apart from the status of a final-year medical student and consent to participate. Additionally, we took several steps to ensure the trustworthiness of our study, including the involvement of two researchers during data analysis, following the established qualitative approach and standards for reporting qualitative research, and inviting authors with diversified professional and educational experiences.

## Conclusions

Following a theoretical framework provided by the theory of planned behavior, the study attempted to evaluate the behavioral intentions of medical students to work in an interprofessional collaborative practice after finishing their studies, along with factors influencing it – attitudes, subjective norms, and perceived behavioral control. The attitudes presented by medical students included both positive, like better patient care, comfort and safety of work, learning and development opportunities, and negative examples, like the fear of conflicts, losing authority, and mistreatment. In the case of subjective norms, students listed the following sources of social pressure to perform or cease from performing the behavior: their peers, other physicians, representatives of other medical professions, patients, and managing bodies. Perceived behavioral control comprised such elements as limited occasions for contacts and interprofessional learning during the studies, existing stereotypes and prejudices, legal regulations and systemic solutions, organizational aspects, and existing relations at the ward. Taking all of the above factors into consideration, it seems that Polish medical students generally hold positive views on interprofessional collaboration, however, they also notice some of its risks and negative aspects. They also feel generally positive social pressure to get involved in interprofessional teams. However, factors responsible for their perceived behavioral control may act as significant barriers in the process. An analysis of the specific factors associated with students’ behavioral intentions seems important, as it allows for diagnosing the current situation and proposing remediation solutions.

## Data Availability

The datasets used and/or analyzed during the current study are available from the corresponding author on reasonable request.

## Abbreviations

TPB: Theory of planned behavior
IPC: Interprofessional collaboration
